# Considerations for Optimizing Warfighter Psychological Health with a Research-Based Flavonoid Approach: A Review

**DOI:** 10.3390/nu15051204

**Published:** 2023-02-27

**Authors:** Tanisha L. Currie, Marguerite M. Engler, Victor Krauthamer, Jonathan M. Scott, Patricia A. Deuster, Thomas P. Flagg

**Affiliations:** 1 Graduate School of Nursing, Uniformed Services University of the Health Sciences, Bethesda, MD 20814, USA; 2 Department of Biomedical Engineering, George Washington University, Washington, DC 20052, USA; 3 Consortium for Health and Military Performance, Department of Military and Emergency Medicine, Uniformed Services University of the Health Sciences, Bethesda, MD 20814, USA; 4 Department of Anatomy, Physiology, and Genetics, Uniformed Services University of the Health Sciences, Bethesda, MD 20814, USA

**Keywords:** fruit extracts, military health, cognitive health, mental health, monoamine hypothesis, bioactive ingredients, cyanidin 3-glucoside

## Abstract

Optimal nutrition is imperative for psychological health. Oxidative stress and inflammation are underlying etiologies for alterations in psychological health. Warfighters are at risk of health concerns such as depression due to increased stress in austere environments and family separation while deployed. Over the last decade, research has demonstrated the health benefits of flavonoids found in fruits and berries. Berry flavonoids have potent antioxidant and anti-inflammatory properties by inhibiting oxidative stress and inflammation. In this review, the promising effects of various berries rich in bioactive flavonoids are examined. By inhibiting oxidative stress, berry flavonoids have the potential to modulate brain, cardiovascular, and intestinal health. There is a critical need for targeted interventions to address psychological health concerns within the warfighter population, and a berry flavonoid-rich diet and/or berry flavonoid dietary supplement intervention may prove beneficial as an adjunctive therapy. Structured searches of the literature were performed in the PubMed, CINAHL, and EMBASE databases using predetermined keywords. This review focuses on berry flavonoids’ critical and fundamental bioactive properties and their potential effects on psychological health in investigations utilizing cell, animal, and human model systems.

## 1. Introduction

Depression is the fourth leading cause of disability, impacting more than 264 million people globally [1]. The COVID-19 pandemic also increased rates worldwide, with reports of a rise in depressive symptoms in American adults [2,3]. Economically, the U.S. spends approximately $1 trillion on mental health and treatments [1]. When left untreated, depression can lead to suicidal ideation and attempts [4,5]. 

Many occupational groups, including law enforcement, healthcare and social worker professionals, artists, academia and administration, food service industries, and the government, are impacted by depression [6]. The highly trained and agile warfighter community is a group that embodies strength and resiliency; however, this community is not immune to the wider societal issues of depression and other mental health disorders, and it has suffered rising rates of depression and suicide [7]. The Armed Forces are consistently faced with the challenges of high-stress work environments and multiple deployments, which have led to high incidences of post-traumatic stress disorder (PTSD), major depressive disorder (MDD), substance abuse, obesity and being overweight, and other comorbid illnesses [8]. According to the Health of the Force report, 18% of warfighters were obese, and 15% of this vulnerable population had at least one behavioral health diagnosis [9]. Depression is prevalent in the warfighter population due to the intense performance demands and requirement to stay operationally fit and ready [10,11]. In a recent Armed Forces surveillance report, 853,060 active component service members were diagnosed with at least one mental health disorder, including depression [12]. 

Mental health and well-being can be enhanced by improving mood [13]. Mood can be described as an actual psychological state that can arise in response to an event [14]. The impact of mood is profound and can shape how things are perceived to influence behavioral, mental, and physical health [13,14]. There are two components of mood: Positive Affect (PA) and Negative Affect (NA). PA is the extent to which a person feels enthusiastic, vigorous, and alert [15], whereas NA is the extent to which a person feels distressed, angry, nervous, or depressed [15]. Depression, a state of low mood, lack of vigor, and diminished quality of life [16], is one characteristic of mood that affects a large proportion of the population with cardiovascular disease.

Currently, cognitive-based therapies and pharmacological treatments address negative moods and depression. Pharmacological treatments may be limited in their effectiveness, as their uptake into the brain may be restricted [17]. Additionally, antidepressant pharmacological therapies such as monoamine oxidase inhibitors (MAOIs), selective serotonin reuptake inhibitors (SSRIs), and serotonin and norepinephrine reuptake inhibitors (SNRIs) often have side effects, such as weight gain, insomnia, hypertension, and stroke in some individuals [18,19]. Therefore, novel and safe solutions are needed as preventive and adjunctive therapies to reduce depression and improve mood.

Mounting evidence suggests that nutrition and, in particular, foods containing flavonoids can affect symptoms of depression [10,20,21]. Flavonoids are a diverse group of secondary metabolites produced by plants that contribute to pigmentation and protect against pathogens [22]. They are commonly found in food and beverages, such as fruit, vegetables, tea, red wine, and chocolate [22]. Flavonoids can be divided into five subclasses: flavanones, isoflavones, flavanols, flavan-3-ols, and anthocyanins [23]. We recently showed that berry extracts inhibit oxidative stress in both cardiomyocyte and microglial cell cultures [24], suggesting that flavonoids derived from plants may be a promising approach to scavenge free radicals and decrease oxidative stress to reduce cell injury [25]. Here, we review recent studies that illustrate the potential promise of berry-derived flavonoids to improve psychological health.

## 2. Data Collection and Database Construction

The following databases were searched on 21 January 2020 for relevant articles: PubMed, CINAHL, and EMBASE. The literature search included combinations of keywords and appropriate subject headings for each database to retrieve articles focused on the use of flavonoids such as blackcurrant on mood and depression. Results were limited to those in the English language. The search strategy identified a total of 661 articles, and after the removal of 415 duplicate articles, 246 articles were screened. A total of 86 articles were reviewed, and the most relevant studies were included within the literature review. To ensure each concept was fully represented for blackcurrant, mood, and depression, incorporated terms such as “Ribes nigrum”, “Ribes americanum”, “Black currant”, “blackcurrant”, “Grossulariaceae”, “mood”, “depression”, and “depressive disorder” were used. 

In an effort to update the most current literature, a second search was conducted on 15 April 2021 using the following databases for relevant articles: PubMed, CINAHL, Web of Science, and EMBASE. The literature search included combinations of keywords and appropriate subject headings for each database that retrieved studies focused on the use of cell models in the investigations with berry flavonoids and their effects on oxidative stress in cardiomyocyte and microglial cell lines. Results were limited to those in English only. To ensure each concept was fully represented for berry flavonoids, terms such as “bilberry”, “cherry”, “blueberry”, “lingonberry”, “elderberry”, “strawberry”, “cranberry”, and “depression and berry flavonoids” were used. For the concept of cell models, the following terms were included: “microglia”, “bv-2”, “cardiac muscle cell”, “rat-atrial-cell”, “cardiac myocyte”, “oxidative stress”, and “inflammation”. The search strategy identified a total of 89 articles. After the removal of 39 duplicate articles that appeared in the search engine results more than once, a total of 50 articles were reviewed, and the most relevant studies were included in the literature review.

## 3. Depression, Oxidative Stress, and Berry Flavonoids

The Monoamine Hypothesis of Depression proposes that altered psychological health is an imbalance of neurotransmitters. Neurotransmitters are instrumental in regulating mood, learning, memory, appetite, sleep, and other executive functions [26]. Decreasing levels of monoamine neurotransmitters can lead to impaired psychological health, such as depression [27]. To mitigate these harmful psychological impacts, standard pharmacological therapies, such as MAOIs, SSRIs, and SNRIs, are typically prescribed by healthcare providers to treat the neurotransmitter imbalances [26]. These pharmacological therapies are often effective; however, dietary substances may interact and interfere with their efficacy or cause adverse effects. An example of this is the “cheese effect” that occurs when tyramine, an ingredient found in cheese, interacts with MAOIs, leading to the acute onset of hypertension that may lead to serotonin accumulation and neurotoxicity [28]. Moreover, these treatments do not address the root cause that led to the imbalance in the first place. 

Depression is also associated with cardiovascular disease [1,29]. A “vascular depression hypothesis” was proposed to underscore the link between depression and vascular diseases, including coronary artery disease, hypertension, and peripheral vascular disease. Alterations in vascular structure and the expression of endothelial cell molecules, such as nitric oxide, have been reported in individuals with depression [30]. This suggests that there may be a common mechanism linking the altered function of these two distinct physiological systems. Oxidative stress that occurs when there is an overproduction of reactive oxygen species (ROS) or an imbalance of antioxidants and ROS [31] emerged as a potential common threat. Inflammation and oxidative stress have independently been linked to both impaired psychological health, such as negative mood [32,33], and cardiovascular disease [1,29]. 

**Figure 1:**
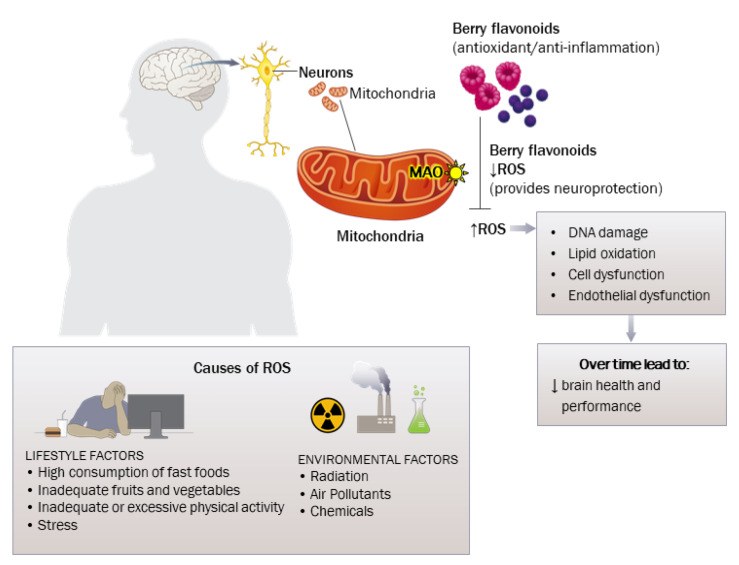
The Effects of Berry Flavonoids on Brain Health.

Lifestyle and environmental or occupational factors can contribute to the development of oxidative stress and lead to impaired neuronal and cardiovascular function (Figure 1). Studies suggest that impaired brain health may be related to the increased production of ROS and induced nitric oxide synthase, elevated cytokines, and increased inflammatory processes [34,35]. Over the last decade, flavonoids have garnered significant interest for their role in bolstering neuroplasticity and dendritic branching, which are necessary processes for neuronal function and connectivity [36]. These bioactive compounds also seem to be neuroprotective, inhibiting apoptosis and downregulating oxidative stress [24,37,38]. Notably, berries such as elderberry, black chokeberry, and blackcurrant are rich in flavonoids called anthocyanins that provide fruits, vegetables, and flowers their color pigments of red, blue, and purple hues [39,40]. Cyanidin 3-glucoside (C3G), a common bioactive ingredient found in berries, is the most frequently detected phenolic compound (out of a total of 203 compounds) in human urine and plasma after the consumption of berries [41]. C3G was detected in 69% of human plasma samples and 58% in urine samples after berry consumption [41]. C3G was also detected in the vascular endothelium and the brain [41]. This suggests that C3G is absorbed and taken up into the body’s tissues, and that it can cross the highly selective blood–brain barrier. Flavonoids can counter the negative cellular effects of oxidative stress and inflammation by modulating cellular signaling pathways [24,38,42]. Although it can vary based on the origin, seasonal conditions, preparations, and primary ingredients, anthocyanins contained in berries generally have a higher antioxidant capacity than other flavonoids [43].

The mechanisms underlying the potential benefit of flavonoids may reflect the combination of many different actions on inflammatory processes, downregulation of oxidative stress, and antioxidant effects. The potential health benefits of berry flavonoids as a nutritional intervention for individual health are illustrated in Figure 1. Excess ROS can damage the mitochondrial energy balance in nervous system, leading to compromised psychological health. Over time, this may also affect brain function and performance. We propose that flavonoid-rich foods can positively affect the warfighter’s psychological health by decreasing ROS and oxidative stress in neural mitochondria.

## 4. Evidence for the Benefits of Berry Flavonoids in the Treatment of Depression

Several studies have investigated the effects of flavonoids on mood as well as cognition [44,45,46]. Depression was found to be increased in older adults who had low intakes of fruits and vegetables [21]. Several studies also demonstrated the benefits of flavonoid-rich diets or beverages in reducing depressive symptoms [47,48,49]. In a double-blind, placebo-controlled crossover study investigating the effect of wild blueberry (WBB) drinks in young adults and children, drinking the WBB drink resulted in significantly higher positive affect scores than sipping the placebo beverage compared to baseline in young adults [50]. A similar improvement in positive affect was observed in children [50]. Similarly, following a diet rich in flavonoids from fruits as well as berries, vegetables, and chocolate for eight weeks was found to decrease depressive symptoms and improve general mental health statuses in mildly hypertensive patients [51]. Moreover, Watson et al. found blackcurrant increases alertness and lowers fatigue in healthy adults [52]. The results of these studies show that flavonoids may improve memory, attention, and cognitive reaction time in both human and animal models [53,54,55]. In a recent article, Kontaogianni et al. randomized participants with hypertension to consume either a low polyphenol diet (LPD), which consisted of two or fewer portions of fruits and vegetables per day with no dark chocolate, or a high polyphenol diet (HPD), which consisted of six portions of fruits and vegetables per day plus dark chocolate, over the course of eight weeks with a washout period [51]. The findings show that the participants within the HPD had reduced depressive symptoms and overall improved well-being in comparison to the control group [51]. Taken together, these studies suggest that berry flavonoids have a promising role in psychological health.

Studies also suggest that the amelioration of depressive-like behaviors correlates with the antioxidant activities of whole berry extracts or constituent flavonoids [51,56,57,58,59]. For example, a recent study showed that Grewia asiatica extracts improved depressive symptoms while increasing antioxidant activity in a rat model [60]. Similarly, blueberry extract improved the antioxidant profile and depression symptoms in a mouse model of depression [57]. In a more recent study, Lycium barbarum berry (Lyc), commonly referred to as Goji berry, was orally administered for four weeks to eight-week-old male rats in an ionizing radiation-induced depression and spatial memory impairment rodent model. Results showed improved cognition, spatial memory, and depression symptoms [61]. Although the Goji berry has potent antioxidant activity, it is unclear whether the positive effects in this study were due to improvement of ROS because it was not assessed. Several studies also demonstrated an antioxidant effect in microglial cells, the resident immune cells in the central nervous system [24,37,62,63]. The potent antioxidative effects of berry flavonoids can be helpful in mitigating harmful processes, and collectively, these studies support the hypothesis that flavonoids and particularly berry extracts can reduce depression through antioxidant effects, act as neuromodulators, and promote cognitive health.

There are other possible mechanisms of the beneficial actions of berry flavonoids. Studies analyzing the relationship between flavonoids and mood suggest that berry flavonoids may modulate monoamine neurotransmitter activity in the CNS [20,32,53]. Acute supplementation with flavonoid-rich blackcurrant was shown to inhibit MAO-A and MAO-B enzymes that suppress dopamine and serotonin levels [64]. Consistent with the positive effect of berry-derived flavonoids on psychological health, blackcurrant extract drinks decreased mental fatigue and improved cognition and mood in healthy adults after a 3-day intervention [64]. This was associated with changes in blood biomarkers, including reduced platelet MAO-B activity. In another study of the effects of blackcurrant juice on mood and physiological biomarkers following treadmill exercise in healthy, sedentary adults, results showed that consuming blackcurrant juice one hour before a walking exercise resulted in a 90% decrease in platelet MAO-B activity (*p* < 0.001) and remained at 25% of baseline by the end [65]. Although this study did not demonstrate significant differences in mood between the blackcurrant and placebo groups, it did provide measures of MAO-B enzyme activity and a brain health marker. In addition, plasma anthocyanin concentrations, the major components in blackcurrant (cyanidin glucoside, delphinidin glucoside, and cyanidin rutinoside), significantly increased in those receiving the treatments compared to those who received the control beverage. Taken together, these studies suggest that blackcurrant or blackcurrant extracts may modulate or downregulate MAO-B activity. 

Alternatively, Hritcu et al. highlighted the relationship of antidepressant-like flavonoids and neuropsychiatric disorders through the suggested modulation of a neurotrophin called brain-derived neurotrophic factor (BDNF) [58]. BDNF plays a critical role in brain health, as it ensures proper neurotransmission, neuronal growth, plasticity, and survival [58,66]. Evidence showed various flavonoids enhanced BDNF expression in the hippocampus of mice models, attenuated oxidative stress and inflammatory properties, mimicked anti-depressant actions, and inhibited monoamine oxidase activities and L-arginine-NO pathways [58]. Earlier reports triangulated a link between decreased BDNF expression, high cortisol levels, and atrophy of the hippocampus with depression [58,66]. Diets possessing appropriate sources of naturally occurring bioactive compounds found in strawberries, grapes, other berry polyphenols, nuts, and spices were shown to play a critical role in hippocampus neurogenesis [67]; conversely, diets that are high in fats and refined sugar lead to decreased hippocampus neurogenesis and decreased BDNF, which is an essential factor in neuroplasticity [67]. Another pathway that has gained greater attention is nuclear factor erythroid 2-related factor 2 (Nrf2). Nrf2 is a nuclear transcription factor, and it plays a significant role in protecting against oxidative stress by regulating antioxidant defense and detoxifying gene expression [68], which contributes to cytoprotection [68]. In a recent review highlighting the role of Nrf2 and natural flavonoid activators by Zuo et al., flavonoids such as curcumin, quercetin, and resveratrol reportedly reduced oxidative stress and inflammation, upregulated glutathione levels in vitro, diminished depressive-like behaviors within a mouse model, and decreased malondialdehyde (MDA) levels, which are a by-product of lipid peroxidation through Nrf2 activation [68]. 

Studies also point to other possible anti-inflammatory and neuroprotective mechanisms. Ebenezer et al. showed that rodents with induced post-traumatic stress disorder (PTSD) demonstrated a significant decrease in proinflammatory cytokines, TLR4, and HMGB1 post-2% blueberry enriched diet through a suggested role of antioxidant defense compared to the control group [69]. Similarly, kaempferol-3-glucoside and narirutin may potentially modulate depressive-like effects in the offspring of a rodent model, whereas a high-sugar diet increased expression of an inflammatory protein marker, TBK1, in the hippocampus [59]. In a study of anxiety-like behavior post-myocardial infarction (MI) in rodents, epigallocatechin-3-gallate (EGCG), which is commonly found in green tea, reduced anxiety-like behavior, decreased inflammatory and apoptosis markers of IL-6 (specifically, caspase 3, 8, and 9 mRNA), and downregulated the STAT3 protein [70]. This study supports a mechanism of action in which EGCG mitigated anxiety-like behavior through suppression of inflammation and apoptosis within the hippocampus of rats post-MI. The observation that flavonoids can affect microglia [24,37,62,63] suggests that the anti-inflammatory activity of berry extracts may provide anti-depressant effects. 

It was also suggested that cardiovascular dysfunction, particularly in older individuals, can lead to the development of depression. The “vascular depression hypothesis” suggests that white matter damage resulting from ischemia may contribute to the onset of depression and cognitive dysfunction [71]. Moreover, rates of depression are elevated following myocardial infarction [72]. Cardiovascular disease continues to be a leading cause of death of Americans [73], and the U.S. active-duty military force is not exempt from its occurrence [9]. One recent study demonstrated that several different classes of anti-hypertensive drugs were associated with a reduced incidence of depression [72]. Several studies have also shown that flavonoids and berry-derived flavonoids improve cardiovascular health [42,62,74,75,76]. Understanding the potential role of flavonoids in cardiovascular health may also help to shed further light on their relationship to psychological health. In a randomized, double-blind, placebo-controlled classic study conducted by Engler et al., young healthy participants during a two-week period were randomized into either a high-flavonoid dark chocolate group or a low-flavonoid dark chocolate group for daily consumption [77]. Cardiovascular and blood marker measures were performed, including investigating the study participants’ flow mediated dilation (FMD) measures. FMD is a non-invasive functional test used to assess arterial blood flow, and it was conducted within this study to measure the effects of dark chocolate consumption in participants randomized to either the high-flavonoid dark chocolate group or the low-flavonoid dark chocolate group. Interestingly, the results show that the high-flavonoid dark chocolate group (containing the higher concentration of epicatechin levels) significantly improved (FMD) levels or increased endothelium-dependent vasodilation in comparison to the control group [77]. Moreover, there were no significant effects on cardiovascular-related blood markers following the study; however, there were significantly higher epicatechin levels found within the high-flavonoid dark chocolate group in comparison to the control group [77]. Either way, this study indicates that the consumption of high-flavonoid dark chocolate with its bioactive compound of epicatechin has health-promoting cardiovascular effects. In another study, the effect of a berry flavonoid, blackcurrant, on vascular health was investigated in a six-week study, measuring FMD and oxidative stress blood markers (i.e., F_2_-isoprostanes) in a healthy adult population that routinely consumed low amounts of flavonoids [78]. Results showed significant increases in FMD, vitamin C levels, and a significant reduction in F_2_-isoprostane levels with 20% blackcurrant juice compared to 6.4% blackcurrant juice and flavored water placebo groups [78]. Similarly, the low (6.4%) blackcurrant group also showed a significant reduction in oxidative stress measures; however, the 20% blackcurrant group demonstrated the greatest cardioprotective effect in reduced oxidative stress and improved FMD measures. Both studies illustrate that moderate to high flavonoid consumption had greater cardiovascular health benefits, such as reduced oxidative stress and improved arterial blood flow outcomes, within these human trial investigations. Additionally, prior work in the literature linked flavonoids and berry flavonoid consumption with improved cardiovascular performance. For example, prior evidence has shown increased cerebral blood flow, enhanced psychological health, and cognitive function following flavonoid consumption [53,79]. Although there is some evidence adding to our scientific knowledge associated with peripheral and central blood flow and its connection to flavonoids and psychological health [79], greater clarity is warranted in understanding the several underlying mechanisms.

Finally, there is an apparent bidirectional association between obesity (defined as a body mass index (BMI) of 30 kg/m^2^ or higher) and depression [80,81,82,83]. Schvey et al. found in their study consisting of 119 active-duty service members of both male and female participants that obesity and being overweight are associated with depressive symptoms, weight stigma, and maladaptive behaviors and eating patterns [84]. Berry flavonoids as a targeted nutritional approach against obesity may provide anti-inflammatory and antioxidant health benefits. For example, in a prospective cohort study consisting of approximately 124,000 men and women that healthcare professionals conducted over 24 years, the results showed that participants who consumed a high flavonoid diet self-reported less weight gain [85]. Although the soluble fiber present in flavonoids has been linked to contributions to weight loss, there may be other underlying mechanisms at play. In reviews by Sandova et al. and Tsuda et al., the potential mechanisms influencing the role of flavonoids in anti-obesity effects include downregulation of lipid accumulation and inhibition of lipogenesis in the liver. Moreover, there was increased browning of white adipose tissue (WAT) and stimulation of brown adipose tissue (BAT), which is important for storing of energy, fat metabolism, and burning calories in the body through induced thermogenesis [86,87]. It is also increasingly recognized that the function of the gut microbiome contributes to overall human health, including brain health [88]. It is postulated that anthocyanins may have inhibitory effects on epithelial cell inflammation, thereby supporting intestinal health through modulation of the gastrointestinal microbiota [89]. Bioactive compounds commonly found in berry flavonoids and teas, such as anthocyanins, quercetin, resveratrol, and EGCG, have shown, individually and collectively, promising actions on increased lipolysis, adiponectin signaling, modulating of the gut microbiota, and influences on body weight reduction [86,87].

## 5. Summary

In summary, the evidence reviewed demonstrates that berries and berry flavonoids can improve psychological health and cognitive function. There has been much compelling research on flavonoids influence on brain aging, neurodegeneration, and memory over the last decade [90,91]. Collectively, the results show that various fruit sources of flavonoids, such as blackcurrant and WBB, may improve mood and decrease fatigue. It is apparent that berries and berry flavonoids possess anti-depressant and anxiolytic effects in both animal and human studies. Despite the increase in research in the field, discerning the mechanisms of the beneficial effects of berry flavonoids remains a difficult challenge.

The complex actions of berry flavonoids that include the antioxidative and cytoprotective effects that may influence cell signaling pathways involved in neurotransmitter breakdown, neuron survival and proliferation, and inflammation have all been implicated as possible mechanisms of action. The association of obesity and cardiovascular disease with depression likely provides an important clue. Cardiovascular disease, obesity, and depression are all associated with persistent inflammation [92,93]. Remaining in a chronic inflammatory state not only disrupts organ dysfunction [92], but it can also instigate depression [94]. Hence, inflammation is a major link to metabolic dysfunction in the form of obesity, and evidence suggests that being in a state of chronic inflammation can trigger further cytokine activity, which can disrupt cellular functioning and overall psychological health. This suggests that targeting oxidative stress and chronic inflammation may be an ideal way to treat depression. Because berries offer a rich source of flavonoids that provide antioxidant and anti-inflammatory activity, this could be an excellent dietary approach.

It should be noted that there are limitations across these studies that must be acknowledged. These include the need for varied study populations and for investigation of additional biomarker endpoints. Further research and clinical trials are needed to explore the potential mechanisms of action of berry flavonoids and their effects on psychological health as well as the optimal dosages for well-being. Another limitation is the consistency of berry composition. The constituent flavonoids vary from berry to berry and from cultivar to cultivar. Thus, more research into the actions of individual bioactive flavonoids may provide a means to develop dietary supplements that provide consistent and reproducible effects.

The increasing rate of depression is a societal problem, and the warfighter population is not immune [12]. This population is heavily burdened with arduous occupational tasks that require a high level of mental acuity, skill, and physical stamina to achieve optimal performance [95]. Identifying beneficial nutritional strategies for the warfighter by leveraging scientific methodologies from the bench to the clinical level may elucidate novel and targeted nutritional interventions and technologies. These new innovations would have the potential to address warfighter injury, recovery, nutrition climate-sensitive rations, and countermeasures for warfighters exposed to extreme hot or cold conditions in austere environments. Using research-based, flavonoid-rich dietary supplements in conjunction with a whole-food, plant-forward diet may also optimize psychological and cognitive health where a whole-food, plant-forward diet is limited. As nutritional science continues to evolve, we must ensure that our nation’s most acceptable human weapon system, our warfighters, is optimally fueled and nourished to meet any demand at a moment’s notice. Strategically, we must also equip and assist our leaders and healthcare professionals through education and bring the science within the ranks to capitalize on warfighter nutrition readiness and psychological health.

The evidence presented here indicates that a research-based dietary supplement and/or increased consumption of a high-flavonoid diet, including berry flavonoids, may prove beneficial in this population. As a flavonoid supplement approach, more rigorous research would need to be conducted concerning product temperature sustainability in austere environments, shelf life, and appropriate flavonoid dosage. Moreover, this may be a portable and feasible solution toward increasing flavonoid intake within this group during austere heat and cold environments. For example, a fruit chew and/or a powdered berry flavonoid drink within warfighter rations or Meals Ready to Eat (MRE) may be of interest within this warfighter population. Providing a berry flavonoid in a portable form in a population in which mental health stigma and psychological risk factors are prevalent is of concern, and it may prove feasible and easy to adapt within the warfighter’s lifestyle.

## 6. Conclusions

Collectively, the findings presented here demonstrate that an assortment of berry flavonoids, such as blackcurrant and their bioactive compounds, and general flavonoids exert various effects with improvements in mood, depression, anxiety, cognitive performance, and overall psychological health. Further research is needed to better understand the varied effects of dietary flavonoids, but evidence supports the conclusion that increasing flavonoid consumption may be an adjunctive therapy for improved psychological health in the warfighter population.
